# Altered affective response to exercise is changed after moderate aerobic exercise training in migraine

**DOI:** 10.1186/1129-2377-14-S1-P140

**Published:** 2013-02-21

**Authors:** A Belitardo de Oliveira, R Teixeira Ribeiro, Carvalho D Souza, MT Mello, S Tufik, MF Prieto Peres

**Affiliations:** 1 Universidade Federal de São Paulo - Brazil, Brazil; 2 Universidade Federal de São Paulo, Brazil

## Introduction

Exercise is known to elicit affective response[1]. However, it is not known how this response behaves in migraine patients. This study is a case report of a young woman with migraine (M) according to ICHD-II. We aimed to verify the effect of 4 weeks of moderate aerobic exercise training (AET) on affective response and M clinical outcomes.

## Methods

AET protocol consisted in treadmill exercise performed 3 times a week for 4 weeks, the intensity of exercise was set at 40-60% of oxygen uptake reserve (VO2R) and duration was 30 min each session[2]. Affective response was measured by the Feeling Scale (FS) of Hardy & Hejeski (1989)[1] at first and last sessions. M clinical outcomes were recorded in a diary.

Results Baseline M-frequency = 13/month (including 5 with disabling intensity). For the 4-weeks AET protocol, M-frequency = 9/month (including 3 with disabling intensity). At first session of aerobic exercise the FS indices measured at moments before, during and after the exercise was 3, 3 and -1(3 = “Well” and -1 = “Bad”), respectively. In the last exercise session the FS indices were 3, 3 and 4(4 = “Very well”) for the correspondent moments.

## Conclusions

These results showed a negative emotional response with exercise and that it was reversed after 4-week of moderate AET. Further research is needed to investigate if these results reproduce in a more representative sample and a longer AET protocol and what neuropsychological mechanisms underlie this behaviour.
